# Towards a Comprehensive Conceptual Framework of Active Travel Behavior: a Review and Synthesis of Published Frameworks

**DOI:** 10.1007/s40572-017-0149-9

**Published:** 2017-07-13

**Authors:** Thomas Götschi, Audrey de Nazelle, Christian Brand, Regine Gerike, on behalf of the PASTA Consortium, B. Alasya, E. Anaya, I. Avila-Palencia, D. Banister, I. Bartana, F. Benvenuti, F. Boschetti, C. Brand, J. Buekers, L. Carniel, G. Carrasco Turigas, A. Castro, M. Cianfano, A. Clark, T. Cole-Hunter, V. Copley, P. De Boever, A. de Nazelle, C. Dimajo, E. Dons, M. Duran, U. Eriksson, H. Franzen, M. Gaupp-Berghausen, R. Gerike, R. Girmenia, T. Götschi, F. Hartmann, F. Iacorossi, L. Int Panis, S. Kahlmeier, H. Khreis, M. Laeremans, T. Martinez, M. Meschik, P. Michelle, P. Muehlmann, N. Mueller, M. Nieuwenhuijsen, A. Nilsson, F. Nussio, J.P. Orjuela Mendoza, S. Pisanti, J. Porcel, F. Racioppi, E. Raser, S. Riegler, H. Robrecht, D. Rojas Rueda, C. Rothballer, J. Sanchez, A. Schaller, R. Schuthof, C. Schweizer, A. Sillero, L. Smidfeltrosqvist, G. Spezzano, A. Standaert, E. Stigell, M. Surace, T. Uhlmann, K. Vancluysen, S. Wegener, H. Wennberg, G. Willis, J. Witzell, V. Zeuschner

**Affiliations:** 1 Epidemiology, Biostatistics and Prevention Institute, University of Zurich, Zurich, Switzerland; 2 Centre for Environmental Policy, Imperial College London, London, UK; 3 Transport Studies Unit, University of Oxford, Oxford, UK; 4 Institute of Transport Planning and Road Traffic, Technische Universität Dresden, Dresden, Germany

**Keywords:** Walking, Cycling, Logic model, Pathway diagram, Behavior theories, Determinants

## Abstract

**Purpose of Review:**

This paper reviews the use of conceptual frameworks in research on active travel, such as walking and cycling. Generic framework features and a wide range of contents are identified and synthesized into a comprehensive framework of active travel behavior, as part of the Physical Activity through Sustainable Transport Approaches project (PASTA). PASTA is a European multinational, interdisciplinary research project on active travel and health.

**Recent Findings:**

Along with an exponential growth in active travel research, a growing number of conceptual frameworks has been published since the early 2000s. Earlier frameworks are simpler and emphasize the distinction of environmental vs. individual factors, while more recently several studies have integrated travel behavior theories more thoroughly.

**Summary:**

Based on the reviewed frameworks and various behavioral theories, we propose the comprehensive PASTA conceptual framework of active travel behavior. We discuss how it can guide future research, such as data collection, data analysis, and modeling of active travel behavior, and present some examples from the PASTA project.

**Electronic supplementary material:**

The online version of this article (doi:10.1007/s40572-017-0149-9) contains supplementary material, which is available to authorized users.

## Introduction

Sustainable transport modes, and in particular walking and cycling, have gained growing interest by decision makers, planners, and the general public as potential solutions to challenges rooted in urban transport, including environmental, economic, and health issues [1,2]. This trend is also reflected in an exponentially growing body of research addressing a wide range of aspects of active travel, including identifying and quantifying determinants of active travel behavior [3], assessing the effectiveness and sustainability of measures to promote it [4–6], understanding and remedying safety related issues [7], developing methods to measure or survey active travel [8], and assessing effects and impacts of active travel on travel, health, and environmental outcomes through various pathways [9–11].

Studies from various fields, such as transport and health, have repeatedly identified and confirmed the role of specific determinants of walking and cycling and regularly presented quantitative effect estimates [12]. However, most studies concentrate on a particular domain of influence, such as the policy context, the built environment, the social environment, or personal and trip attributes. Often, they are also limited by their specific topical perspectives, such as transport issues or public health. “Transport studies” tend to ignore health both as a motivation and as an outcome of active travel, while “health studies” often ignore the role of competing modes of transport. While taken together the whole body of knowledge does paint a fairly extensive qualitative picture of determinants of active travel, it remains challenging to build robust comprehensive models combining quantitative estimates. Namely, coefficients derived from different studies are not adjusted for each other, they may be based on different scales and definitions, and they tend to stem from different contexts and populations. A more holistic quantitative understanding of determinants of active travel, however, would help answer some of the most practice-relevant questions. In particular, it would provide more robust evidence to identify effective measures and policies and rationalize how these are prioritized. One possible application would be to better integrate walking and cycling into travel demand models, which to date tend to represent active travel poorly [13,14].

To develop a more holistic quantitative understanding of determinants of active travel and how it could be promoted, larger more comprehensive studies with a broad scope need to be conducted. We argue that for such endeavors to succeed, it is crucial to acquire the best possible conceptual understanding of the relationships between relevant determinants and active travel behavior and potential confounders and mediators a priori.

This paper thus aims to (1) review the use of conceptual frameworks in active travel research, and, based on these frameworks, (2) to propose a comprehensive framework that covers the abovementioned domains and can guide future research to observe, explain, and model active travel behavior. We aim to first systematically identify and describe key features of conceptual frameworks for active travel and then apply these in a novel, systematic and comprehensive framework developed within the scope of the Physical Activity through Sustainable Transport Approaches (PASTA) project [15•], a multinational, interdisciplinary research project on active travel and health. The so-called PASTA framework was initially developed and used to determine contents of the longitudinal PASTA survey, a broad data collection effort about active travel and physical activity, their determinants, and associated crash risks [16]. While a visualization of such a framework could hardly be comprehensive with regards to topical scope, level of detail, or methodological issues for all active travel related research, we aim to combine as many concepts as possible identified by previous work; we aim to do this systematically; and we aim to provide generalizable guidance on how to be more systematic in developing frameworks for active travel related research. In the final section, we discuss the value of such efforts and provide examples of how the PASTA framework can guide specific research efforts.

## Methods

### Literature Review

A systematic effort was taken to comprehensively identify conceptual frameworks for active travel published in the scientific literature. However, despite clearly defined search terms, multiple steps to identify relevant publications, and systematic summaries of identified publications, this review remains exploratory, because “conceptual framework” is a loosely defined term and presumably some researchers may use a framework as a working tool without presenting or referring to it in publications or specifying which theories they are built on.

Systematic literature database searches of PubMed and the Transport Research International Documentation (TRID) database were complemented with systematic scans of references listed in publications identified as relevant (see Appendix 1 for search terms and hits.)

Identified hits were inspected with regards to relevance for the objectives of this review using a standardized categorization form filled out by at least two independent reviewers for each study. The form captured the scope of the reviewed publications, the purpose of the framework, the audience it was aimed for, its novelty, underlying theories, and area of research. Particular focus was put on unique or novel concepts and visual framework features that could contribute towards a more comprehensive framework.

The Results section describes the range of frameworks deemed within scope of the review and presents an overview (Table 1) and summaries of the most relevant publications.

**Table 1:**
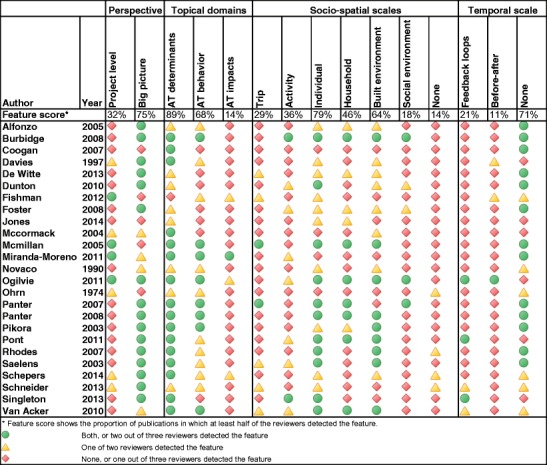
Frequency of framework features among the 26 publications presenting new conceptual contents

*Feature score reflects the degree of reviewers detecting a feature among reviewed studies

### Synthesis of Reviewed Frameworks and Development of the PASTA Conceptual Framework for Active Travel

Based on the identified conceptual frameworks of active travel, the study of behavioral theories, and our understanding of key issues of active travel behavior, we developed a more comprehensive conceptual framework for active travel behavior. The term comprehensive reflects our intention to integrate research-relevant aspects of active travel including various topical domains and structural features in a compact and well-balanced way. To facilitate readability, we present a simplified version in the main text and refer to Appendix 3 for a detailed version (for additional versions, see the supplemental materials available in https://www.researchgate.net/profile/Thomas_Goetschi/publications).

Specifically, the framework aims to address aspects of relevance to the PASTA study and presumably other research projects, namely, guidance to identify data needs, data sources, and collection methods; data hierarchies, such as clusters or levels of aggregation; the integration of transport and health perspectives with a focus on active travel; and importance of context in measuring and evaluating active travel interventions. Finally, the framework allows testing elements of established theories and latest thinking in (active) travel behavior research.

## Results

### Overview of the Identified Frameworks

The literature search yielded over 200 hits in PubMed and TRID. After scanning abstract and titles for relevance, reviewing references, and adding some publications identified previously, 65 publications were kept for further review. Appendix 1 provides an overview of the selection process.

The main scope of two thirds of these publications was on active travel behavior [*48/65*], while about one third also focused on physical activity [*23/65*] or safety [*8/65*]. In terms of audience, the publications appeared to target about equally transport researcher, health researchers, and planners, whereas health professionals, policy makers, and advocates seemed somewhat less represented.

About two thirds of the identified publications were considered to provide and discuss conceptual frameworks [*43/65*], in the sense of aiming to illustrate causal, temporal, spatial, or hierarchical relationships between active travel outcomes and factors that explain these. Around 10 to 15 papers instead presented frameworks for planning, data collection, modeling, designing interventions, or conducting evaluations.

About half of all publications presented at least in parts new contents [*36/65*], whereas the others referred to previously published frameworks [*29/65*] or theories [*10/65*]. Publications not providing substantial new conceptual contributions most often referred to existing frameworks, such as the socio-ecological model [17], or relevant theories, such as the theory of planned behavior [18].

Among those frameworks considered conceptual and new [*N =* 26] (see Table 1), about two thirds took a more general big picture perspective [*21/26*], whereas in one third the framework guided a specific project [*9/26*]. The majority covered the domains of active travel behavior [*18/26*] and its determinants [*24/26*], whereas four frameworks were concerned with impacts of active travel. Frameworks on active travel behavior split about equally between walking [*12/18*] and cycling [*11/18*], with almost half of all frameworks covering both modes. Most publications centered around the individual level [*22/26*], while many additionally considered the built environment [*18/26*] and households [*13/26*] as distinct structural levels. Only eight studies treated trips as a separate level. Temporal structures were absent in most frameworks [*20/26*] with few exceptions indicating feedback loops [*6/26*] or changes over time (before/after) [*3/26*]. About three quarters provided illustrations of the frameworks.

In terms of determinants of active travel, built environment [*23/26*], infrastructure measures [*21/26*], psychological [*17/26*] and socio-demographic factors [*19/26*] obtained about equal attention. Among the frameworks addressing travel behavior [*18/26*]*,* most described active travel in generic terms, such as walking [*13/18*], cycling [*12/18*], or even physical activity [*9/18*], but several frameworks were more nuanced by investigating different modes of travel, journey purposes (including walking or cycling for recreation or transport), and various quantitative measures, like frequency or duration. The few frameworks that address impacts of active travel [4] covered environmental (e.g., carbon emissions), health, and safety outcomes.

In the following section, we review a selection of frameworks considered most appropriate in providing distinct features to the development of a more comprehensive framework of active travel behavior. These features are briefly highlighted. Illustrations of some of these frameworks can be found in the supplemental materials available in https://www.researchgate.net/profile/Thomas_Goetschi/publications.

### Review of Selected Frameworks

Pikora et al. [19] and Saelens et al. [20] were among the earliest publications showing conceptual frameworks for active travel behavior. Their frameworks are imbedded in a socio-ecological model [17], focusing on different layers of environmental (ecological) and individual (socio-psychological) factors explaining active travel outcomes. Such models are widely applied in health behavioral science, as reviewed for example by Sallis et al. [21]; they describe the role of the combined effects of psychosocial and environmental variables (i.e., community, policy) to explain physical activity. They imply that a combination of environmental and personal level factors will best explain behavior and that addressing both—or multiple—domains will allow for the development of most effective interventions. Saelens’ model, derived from a review of planning literature, distinguishes active travel for transport vs. recreation, as well as stronger and weaker links between factors.

Ogilvie et al. [4,22] presented a framework with the purpose to measure and evaluate changes in active travel and physical activity behavior resulting from physical infrastructure interventions as part of the longitudinal cohort study “iConnect” [23]. The iConnect model builds on the framework presented by Saelens et al. [20], which is expanded beyond active travel to include overall travel behavior, overall physical activity, and imputed impacts such as carbon emissions from motorized travel [4]. In addition, psychosocial factors, such as habit and social norms based on the extended theory of planned behavior [18,24], as well as social environment factors are introduced as key factors affecting behavior change. The framework applies Pawson and Tilley’s “realistic evaluation” framework [25] that postulates to distinguish and specify “contexts, mechanisms, and outcomes” (so-called CMO configurations) in order to evaluate how interventions work, for whom, and in what circumstances.

Panter et al. [26] built on a framework on school travel proposed by Macmillan et al. [27,28] and Pikora’s socio-ecological model for physical activity [19]. Focusing on active travel to school, they distinguished environmental and individual factors, paying particular attention to the interplay of parents’ and youth’s perceptions affecting mode choice for school travel. Within environmental factors, neighborhoods, destinations, and routes are distinguished, expanding the socio-ecological structure to include travel-specific elements. Also on the topic of parent youth relations when it comes to mode choice, Pont et al. [29] provide a framework most notable for its depiction of the parent child relation, as one of few examples illustrating a process over time.

Based on an extensive literature review, Burbidge and Goulias [30] proposed a conceptual framework mainly combining elements of the theory of planned behavior [18] and decision field theory [31], which they complemented with additional factors identified from the literature, such as infrastructure and residential location selection. At the core is a mode choice process, which is influenced by personal attributes, infrastructure and environment, time allocation, and various related factors. The choice process itself, however, is not explored in detail.

Schneider [32] explored the choice process in more detail proposing a theory of routine mode choice decisions in the wider context of policies attempting to shift trips from motorized to non-motorized modes. The theory suggests a five-step mode choice process, consisting of (1) awareness and availability of possible mode choices, (2) safety and security, (3) convenience and cost, (4) enjoyment, which then determine tradeoffs between the possible mode choices, and finally (5) habit, which reinforces earlier choices. Socio-demographic characteristics serve as moderators of these concepts. The theory builds on numerous insights from travel behavior research and psychology [18,33,34] and is substantiated with empirical evidence from qualitative interviews of San Francisco Bay Area residents.

Based on Maslow’s theory of human motivation [35], Alfonzo [36] presents a similar hierarchy of walking needs, where the individual first assesses feasibility, then accessibility, safety, comfort, and “pleasurability.” This is linked with moderating processes defined by life-cycle circumstances to determine outcomes. The life-cycle circumstances themselves include regional, group, and individual level factors such as climate, culture, and psychological factors, respectively (among others).

Singleton [37••,^38^] proposed a framework based on an extensive review of travel behavior theories with the intention to improve direct applicability to active travel forecasting models. Its travel decision-making process also includes a hierarchy of travel needs, which are mediated by individual perceptions and decision rules (e.g., how a shorter trip distance is weighted against a higher crash risk [39–41]). At the start of the process is an activity, which results in a travel demand, or motivation, or desire to travel.

Martin et al. [42] explore the choice process with a specific focus on the mechanisms of policies. The study reviewed publications on policies that provide financial incentives to promote active travel, illustrating traditional economics (i.e., utility maximization, [39]) and psychological behavior theories (including behavioral economics) in terms of specific choice formulations and examples of policies addressing these [43,44].

In their extensive review of travel behavior and psychological theories, Van Acker et al. [34] synthesize numerous concepts of relevance to active travel. Their conceptual model emphasizes the distinction between reasoned influences on behavior, such as perceptions, preferences, and attitudes, and unreasoned influences driven by habits and impulsiveness. Feedback loops indicate the possibility of changes over time. Behavior in this framework is depicted as set of levels that range from the most short-term travel behavior to activity and locational behavior, all the way to lifestyle. Individuals’ behavior is further determined by opportunities and constraints, which present themselves at the individual level, as well as through the social and spatial environment.

In a similarly broad review of mode choice literature, De Witte et al. [45•] emphasize an interdisciplinary perspective, identifying and distinguishing determinants by rationalist (i.e., journey characteristics), socio-geographical (i.e., spatial indicators), and socio-demographic domains, which in combination with socio-psychological factors determine mode choice.

In addition, numerous studies have published frameworks of relevance in the context of active travel that are not directly concerned with active travel behavior, such as safety [46,47•,^48^], types of cyclists [49], or physical activity [50], or do not specifically address active travel, such as MINDSPACE, a framework of how public policy influences behavior [43]. Further, there is abundant literature on travel behavior in general, and numerous theories have conceptualized it, many of which are reflected in the reviewed active travel frameworks. We refer to others for overviews of relevant theories [34,37••,^51^].

## Synthesis of Results and Development of the PASTA Framework of Active Travel Behavior

### Key Features of Conceptual Frameworks for Active Travel

In this section, we synthesize and discuss key features of identified and reviewed conceptual frameworks for active travel and related theories and describe how we develop these to build the comprehensive PASTA conceptual framework of active travel behavior (from here on referred to as the PASTA framework). Our aim hereby is to absorb as many relevant concepts encountered in the reviewed frameworks and pertinent theories into a single, systematically structured framework as possible. To do so, we distinguish three features of conceptual frameworks representing active travel behavior:

- Behavioral decision or choice process
- Structural scales and relationships
- Contents and topical domains

### Behavioral decision or choice process

**Fig. 1:**
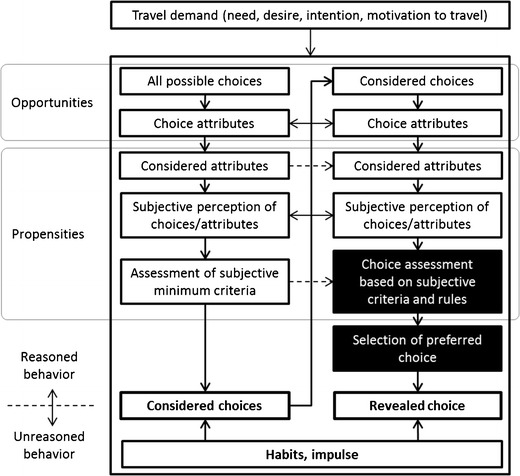
Generic choice process for active travel-related behavioral decisions. The column on the *left* illustrates the process to identify considered choices. From these, one choice is selected through the process in the column on the *right*

Similar to others [30,37••,^38^], we conceptualize the core behavioral process as a demand (or need, desire, intention, or motivation) to travel that is derived from the need or desire to participate in an activity (i.e., work, shop, escort kids to school, visiting friends or family). This triggers a choice process which produces a behavioral decision or response, namely a revealed choice or outcome. (See Fig. 1. For a simplified diagram of the generic process, see supplemental materials available in https://www.researchgate.net/profile/Thomas_Goetschi/publications.)

In the transport context a choice is often discrete, short-term, and trip related (i.e., mode choice, route choice) [39], but it is noteworthy that behavioral outcomes can also be aggregates of many choices over time, such as individual attributes of interest in health research (e.g., minutes of cycling per week or long-term physical activity derived from active travel). Active travel warrants consideration of both perspectives, and in the detailed version of the PASTA framework, we distinguish the two (see Appendix 3). For a systematic listing of cycling related choices and outcomes, see supplemental materials available in https://www.researchgate.net/profile/Thomas_Goetschi/publications.

Activities have their own temporality (rhythm, frequency, ir/regularity) and spatiality (distance vis-à-vis anchor locations (home, primary employer, possibly childcare, school) and spatial variability). Many of these activities cannot be adequately represented as rational choice behavior—they are at best boundedly rational [52] because of constraints on information availability and processing. But even this is a gross simplification as it does not consider the social dimensions of taking part in those activities.

As such, the choice process may very well be too complex to be explained by a single theory or depicted in a simple diagram. Nonetheless, numerous published concepts and theories have provided helpful guidance for research. Similar to several others [26,30,36,37••], we conceptualize the choice process in our framework as the central pathway that takes into account objective and subjective factors or opportunities and propensities [53], respectively, to make a behavioral decision or reveal a choice.

Figure 1 depicts the generic process. At the beginning, a travel demand (or activity) is met with objective opportunities, resulting in many theoretically possible choices, each with their specific attributes. The first of two sub-processes, or iterations (vertical flows in the diagram), identifies a subset of *considered choices*, which then in a second process are assessed to identify the *selected choice*. In reality, these may not be perfectly distinct, may inform each other, or may run in parallel (as indicated by parallel arrows).

When based on reasoned behavior [18,54], as illustrated for example by van Acker [34], these selections are derived from some combination of the objective attributes of the possible choices (or opportunities), such as mode accessibility, trip duration, safety, etc., and a set of subjective factors (or propensities), such as perceptions, attitudes, values, rules, preferences, and norms [12]. In unreasoned behavior, on the other hand, a habit or impulse circumvents the reasoned choice process leading directly to a choice without (much) consideration of other factors [33,34].

We conceptualize the reason-based part of the choice process similar to a utility-based approach assuming utility maximization behavior, as is commonly done in transport modeling [13,37••,^39^,^55^]. However, empirical random utility maximization models are often constrained to a small number of measurable utility factors (i.e., monetary and time cost), which fail to capture the multitude of considerations determining active travel choices (e.g., weather, safety, health benefits, pleasure [56,57]). Moreover, active travel decisions, possibly more so than is the case for motorized travel behavior, may be based on other decision rules than compensatory utility maximization, as pointed out by Singleton and Clifton [37••,^38^]. We therefore conceptualize this part more loosely as a generic *choice assessment*, which compares any sort of subjective value a subject is to gain from a choice over an alternative (black boxes in diagram). This accommodates active travel-specific utility dimensions, such as perceptions of safety, or expected health benefits, which are relatively more important for active travel than for motorized travel, but also irrational preferences, fears, or principles (i.e., non-compensatory rules [37••]). It is important to point out that the choice assessment is limited by the factors a subject actually considers and its perception or information of these, which may be “highly subjective” or incomplete (i.e., bounded rationality [58]).

While habit and impulsiveness complicate the link between need and revealed behavior, their importance warrants inclusion in our framework. We therefore keep non-reasoned behaviors, like sticking to a habit [59], following an impulse to try something new, or taking risk against better knowledge, separate from the reasoned choice assessment. However, it seems plausible that both the reasoned and unreasoned pathways could influence the same decision.

The way the generic choice process manifests in a specific situation depends on numerous environmental and personal factors. We treat these as determinants outside of the generic choice process.

### Structural scales and relationships

The distinction of structural scales, such as hierarchies or clustering between factors, is of practical relevance for data collection and analysis, the validity of an analysis, and various other practical and methodological aspects (e.g., intervention design). Among the reviewed frameworks, the most common distinction is between environmental and personal factors, but further visualizations of spatial, social, or temporal scales have been used. The degree to which frameworks distinguish such structures varies tremendously. Aligning frameworks along structural scales seems particularly helpful when the distinction serves some specific purpose (e.g., data collection, model structure). We underlay the PASTA framework with a hierarchical *socio-spatial pyramid* (see Appendix 2, Fig. A2), building on the often-referenced socio-ecological framework [19,21,22], which we expand to include travel-specific sub-individual layers, such as activity (leading to one or multiple trips), trip origin and destination, departure time, route, and mode of travel. These structures equally guide where we present determinants and outcomes.

**Fig. 2:**
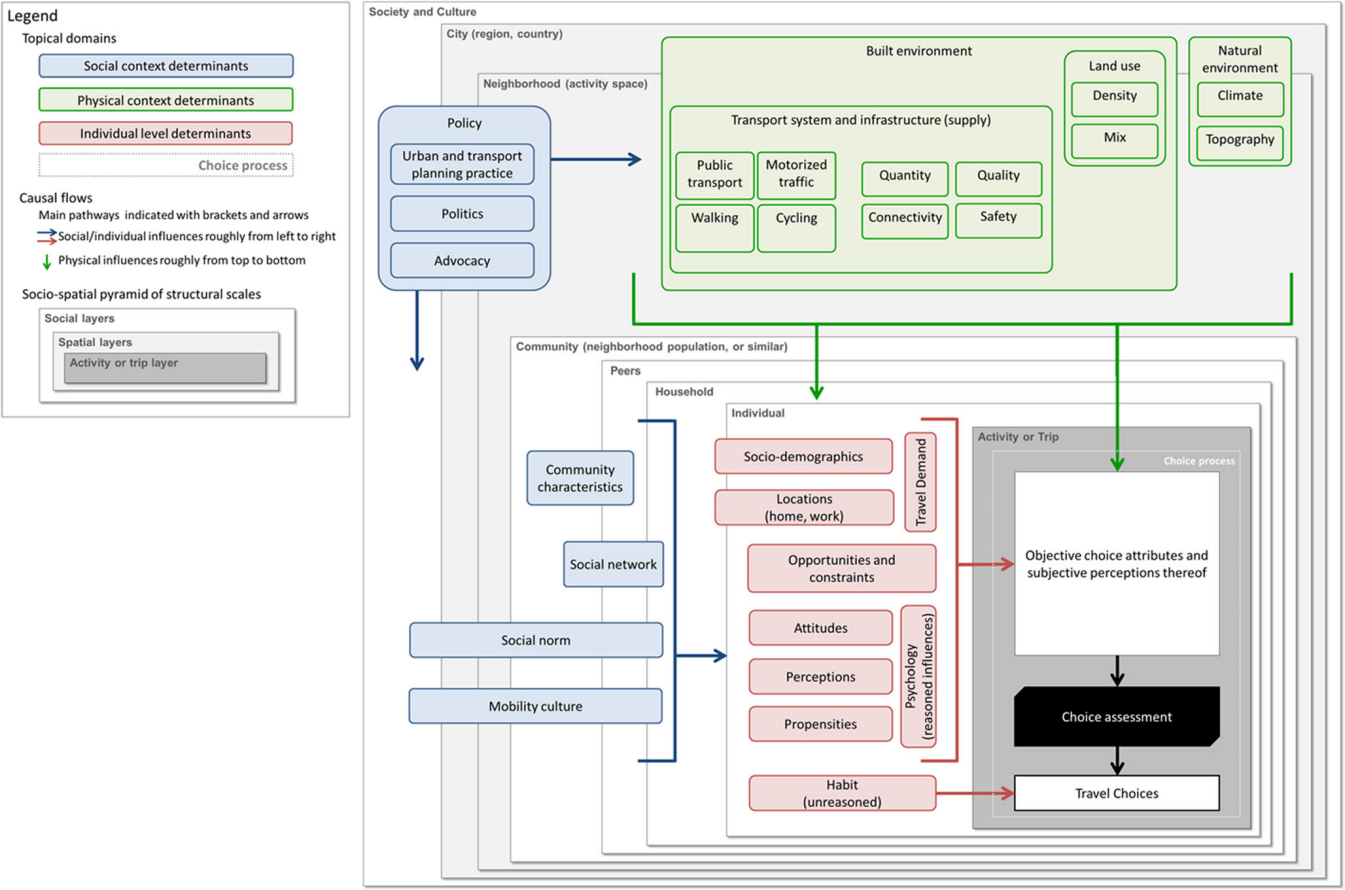
PASTA conceptual framework of active travel behavior. A more detailed version of the framework including a detailed reader’s guide is available in Appendix 3. Additional variations of the framework are available in the supplemental materials in https://www.researchgate.net/profile/Thomas_Goetschi/publications

The generic PASTA framework (Fig. 2) represents a *static view* on active travel behavior as opposed to behavioral *change*. To a large extent, the *temporal scale* is captured within the socio-spatial structures, in the sense that highest resolution layers (i.e., trips) reflect more short-term phenomena, whereas at the larger layers (i.e., society or city), processes take longer (i.e., changes in factors or periods over which factors affect each other [34]). Explicitly visualizing temporality of relationships would be warranted in frameworks focusing on behavior change, such as developments over the life course [49], effects of interventions [22,23], or those depicting behavioral change along a sequence of defined concepts or stages [40,60]. In quasi-static frameworks, feedback loops or cascading concepts are commonly used to indicate temporality [29], and some concepts capture it implicitly (e.g., habit).

In most published frameworks, directional arrows are the instrument of choice to indicate some sort of relationship or association between factors (or concepts). Variations include dashed and bidirectional arrows, sometimes labeled. The usefulness of arrows seems directly dependent on the specificity of factors and the understanding of relationships. In fairly comprehensive frameworks, the sheer number of related factors often prohibits the illustration of all relationships and using arrows without systematic and transparent criteria can become misleading. Alternatively, or additionally, spatial proximity and clustering or overlapping of factors is used. In the PASTA framework, we indicate relationships or influence between factors predominantly by proximity, arrangement, and clustering of factors (i.e., without using arrows). Only key causal flows are suggested by braces and arrows. Further, we aim for consistent directions of logical pathways (i.e., determinants leading to outcomes). Namely we arrange pathways of social factors roughly from left to right and pathways of environmental factors from top to bottom. However, when applied for specific purposes, such as conceptualizing a specific analysis, more specific arrows become useful (see supplemental materials available in https://www.researchgate.net/profile/Thomas_Goetschi/publications.).

### Contents and topical domains

Finally, the above structures are only meaningful when populated with specific, meaningful contents. Comprehensiveness and level of detail are direct tradeoffs and ultimately depend on the specific purpose of a framework but generally impose a challenge for visualization (though to a lesser degree in electronic documents that can be zoomed in, see Appendix 1). We arrange factors by topical domains, such as natural and built environment, socio-demographics, personal propensities, psychological factors, etc. We capture obvious sub-factors in overarching concepts to improve the readability of the framework.

### Practical Applications of the PASTA Framework in Active Travel Research

There are many practical applications of conceptual frameworks. In particular, in the context of conducting a (large) research project, we argue that the consideration of a detailed conceptual framework, and possibly the development of a study-specific version, is crucial. The choice of data sources, data collection methods, and survey contents and when and where to sample are crucial questions in successfully pursuing research objectives. In the PASTA project, the framework served as a valuable cross-reference to assure the inclusion of as many of the most relevant determinants and potential confounding variables as possible, while at the same time keeping topical areas well-balanced and user burden in check (adapted framework versions are available in the supplemental materials available in https://www.researchgate.net/profile/Thomas_Goetschi/publications).

The challenges of comprehensively assessing covariates and mediating factors are even more aggravated in research on changes in active travel behavior over time. Effects of interventions are typically small, requiring utmost attention to control of confounders and modifiers. A comprehensive framework is a helpful tool to identify these and anticipate their roles [60]. In PASTA, the framework guides the evaluation of key measures to promote walking and cycling, selected across the participating cities. The framework allows us to identify the critical putative causal pathways by which we believe the active travel measures are likely to work and as such informed survey contents and how we analyze these. We illustrate an example application highlighting the effects of the construction of high quality cycle highways in London and Antwerp and through which pathways they affect cycling behavior and in particular “stages of change” [40] (supplemental materials available in https://www.researchgate.net/profile/Thomas_Goetschi/publications). Simplified, cycle highways affect regular cyclists through the provision of better routes (more direct, pleasant, and safe), while for infrequent or potential cyclists, the main pathway presumably is through an improvement in perceived safety, which may help them pursue their intention to bike more or pick up cycling.

Finally, the framework can also be helpful to inform sampling schemes. In PASTA, relatively stable personal factors, like general health or attitudes, were surveyed only once as part of a baseline questionnaire, whereas the more short-term and temporally variable factors like travel or physical activity behavior were surveyed in frequent follow-up questionnaires every 2 weeks.

## Conclusions

Conceptual frameworks have been used to visualize a wide range of aspects of active travel behavior. Despite a remarkable diversity in illustrations, several common features could be identified. To date, the PASTA framework provides a first-of-its-kind effort to systematically combine behavioral concepts, structural features, and a large number of determinants identified in the literature as part of a single, comprehensive framework to inform future works. In PASTA, the framework provided valuable guidance in developing survey contents and study design, and in particular, for the combination of research approaches from the transport and health disciplines on how to best measure active travel and related factors. We conclude that the systematic development and use of a conceptual framework can provide invaluable support to design and conduct more elaborate and comprehensive active travel studies need to address key research gaps.

## Electronic supplementary material

ESM 1 (DOCX 765 kb)
