# Supplementary material for: Towards a Comprehensive Conceptual Framework of Active Travel Behavior: a Review and Synthesis of Published Frameworks
Source: Curr Environ Health Rep. 2017 Jul 13;4(3):286–95. doi: 10.1007/s40572-017-0149-9 (PMC5591356; doi:10.1007/s40572-017-0149-9)
Supplement: Supplementary file 1 — (DOCX 765 kb) [file 40572_2017_149_MOESM1_ESM.docx]

# Appendix

## Literature search terms and search hits

((Active mobility[Title] OR active commute[Title] OR commut*[Title] OR active transport*[Title] OR active travel*[Title] OR non motor*[Title] OR bik*[Title] OR bicycl*[Title] OR cycling[Title] OR cyclist[Title] OR walk*[Title] OR pedestrian[Title] OR "Bicycling"[Mesh] OR "Walking"[Mesh] OR "Walkers"[Mesh]))

**AND**

(framework[Title/Abstract])

**NOT**

(“random walk” OR octane framework OR bicyclic OR bicyclo OR

"Cells"[Mesh] OR plants OR "Plants"[Mesh] OR bacteria OR "Bacteria"[Mesh] OR animals OR aircrafts OR airplanes OR "Aircraft"[Mesh] OR airports OR water OR "Water"[Mesh] OR deserts OR violence OR "Violence"[Mesh] OR "Tropical Medicine"[Mesh] OR "Genetics"[Mesh] OR genetics OR biotechnology OR biology OR "Biology"[Mesh] OR microorganisms OR "Microbiology"[Mesh] OR biotechnology OR "Biotechnology"[Majr] OR DNA OR "DNA"[Mesh] OR enzymes OR "Enzymes"[Mesh] OR surgical OR "Surgical Procedures, Operative"[Mesh] OR clinical OR "Pathology, Clinical"[Mesh] OR diagnosis OR "Diagnosis"[Mesh] OR "Vaccination"[Mesh] OR vaccination)


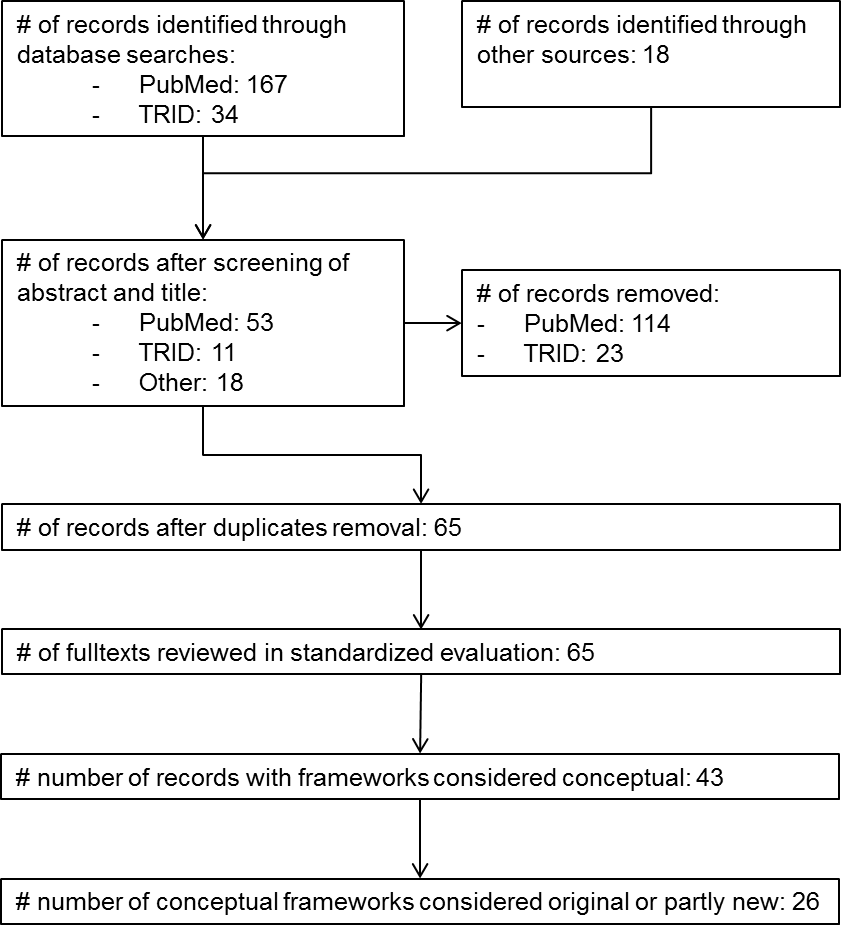


Figure A1. Flow diagram of literature search and review process.

## Socio-spatial pyramid of structural scales in the PASTA conceptual framework of active travel behavior


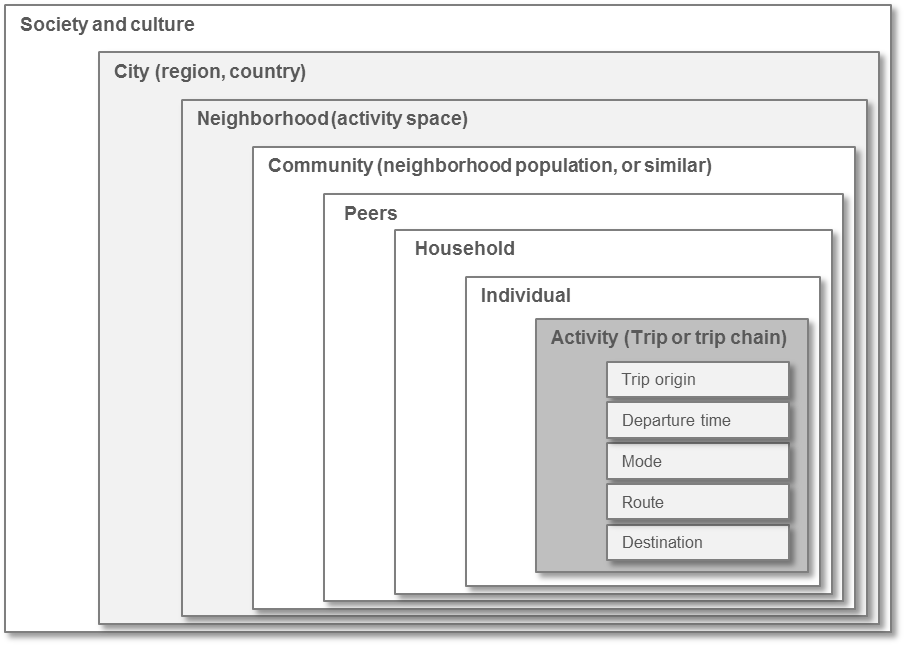


Figure A2. Socio-spatial pyramid of structural scales in the PASTA conceptual framework of active travel behavior, based on the socio-ecological framework (19), expanded to include travel related layers. Social layers are in white, spatial layers in light grey. At the center (or top) in dark grey is the travel-specific sub-individual layer of activity (or trip, or trip chain) which contains the equally ranked domains of origin and destination locations, departure time, route and travel mode. Community refers to a social dimension of the area a subject frequents, whereas neighborhood refers to its physical counterpart.

## Detailed version of the PASTA conceptual framework of active travel behavior

### PASTA Conceptual Framework of Active Travel Behavior


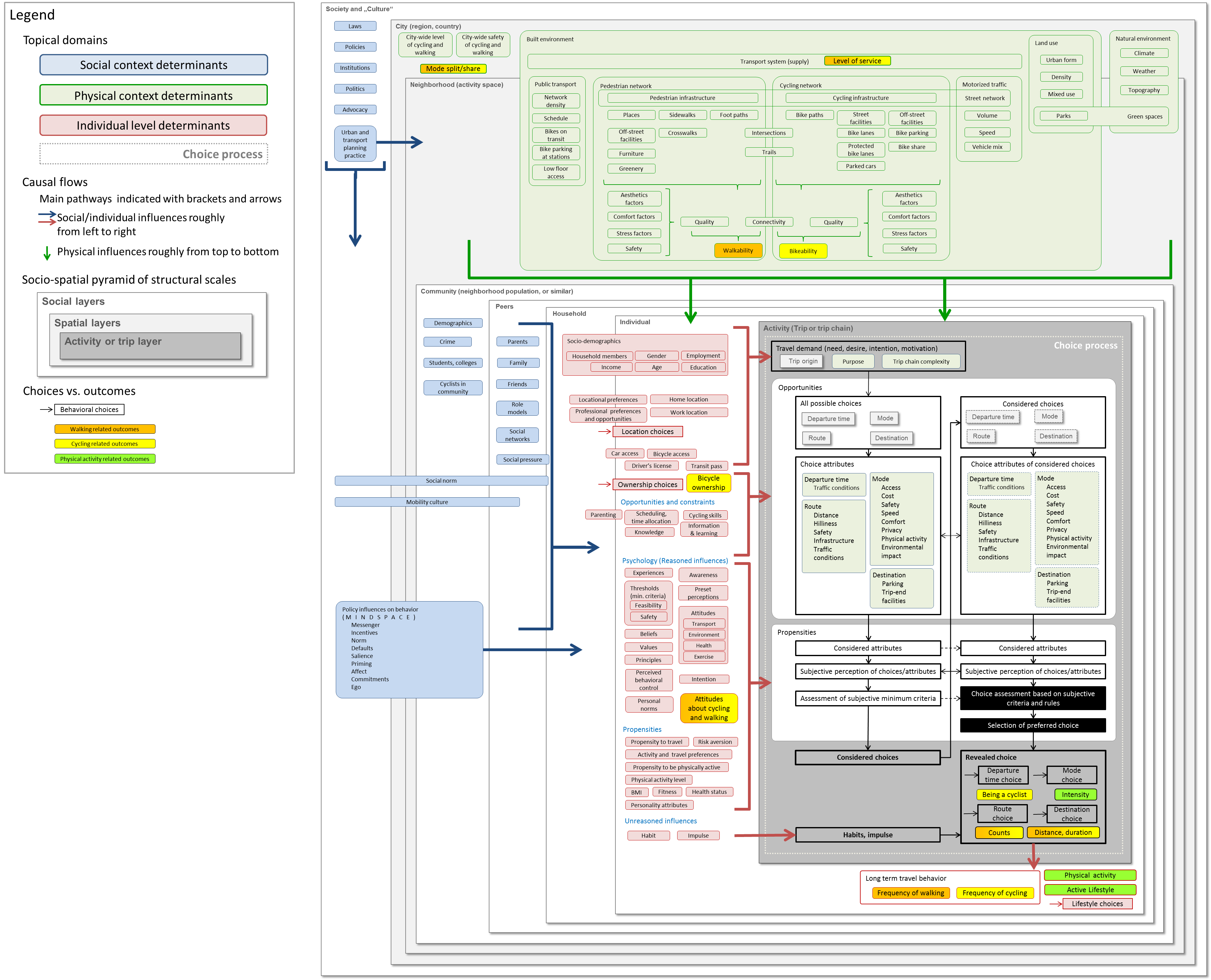


Figure A3. Detailed version of the PASTA conceptual framework of active travel behavior. See user guide (1.3.2) for detailed guidance.

### User guide for the PASTA conceptual framework of active travel behavior

- Topical domains
  - Social determinants of active travel behavior are shown to the left in blue.
  - Physical context (or environmental) determinants are shown on top in green.
  - Determinants at the individual level (socio-demographics, psychology, etc.) are depicted in red.
- Causal flow (direction of logical pathways)
  - Overall flow of causal pathways is roughly from top left to bottom right.
  - Pathways for social and individual factors roughly lead from left to right.
  - Pathways for physical (spatial, environmental) pathways roughly lead from top to bottom.
  - Influences of key domains are indicated with braces and arrows. Proximity of factors further suggests topical or causal relationships.
- Structural scales
  - Social and spatial scales are conceptualized as a hierarchical pyramid (or cluster of frames), with activity as the highest (or most center) layer representing the most short term travel behavior, which is affected, more or less directly, by the underlying layers (i.e. individual, household, etc.). Also see Figure 4.
    - Activity (or trip, or trip chain) is in the dark grey frame, spatial scales are in light grey frames, social scales are in white frames.
  - Activity layer
    - Simplified, this can be thought of as a single trip, but more realistically it addresses activities that require travel. As such, it may also apply to trip chains rather than just a single trip.
    - Choice process
      - The travel choice process is illustrated at the activity (or trip) level.
      - Concepts of immediate relevance to the choice process are illustrated in the dark grey frame.
      - Choice assessment and selection are depicted as black boxes which process choices and their attributes and subjective perceptions thereof by applying subjective choice criteria.
        - Possible choices consist of departure time, mode, route and destination, respectively, each of which comes with its set of attributes.
      - Environmental factors (physical context) which determine available choices and their objective attributes are depicted at the top, in the spatial layers (i.e. neighborhood, city).
      - Factors that determine the considerations of choices and the perceptions of their attributes are depicted to the left, at the individual level.
      - The choice process results in a revealed choice, or set of linked choices.
  - Individual layer
    - Objective factors, which determine a travel need are shown at the top of this box.
    - Subjective or psychological factors, which influence the choice process are shown below.
    - Unreasoned factors, like habit, which circumvent the choice assessment process are shown at the bottom.
    - In the bottom-right corner longer term travel behavioral outcomes are listed.
  - Spatial layers (Neighborhood, city)
    - Neighborhood refers to a geographical space in which most of a subject’s routine travel occurs (excluding long distance travel, like vacations, etc.).
    - City refers to a somewhat larger geographical area including parts which may not be frequented regularly by an individual. This layer includes city-wide factors which may nonetheless affect an individual’s or community’s perceptions of travel choices (i.e. convenience of public transport, or safety of cycling).
    - We depict environmental factors above the individual and activity layers, but point out that environmental factors affect cycling behavior through numerous pathways (i.e. “from all sides”), determining activity attributes and affecting individuals’ perceptions.
    - Built environment
      - We roughly sort factors by hierarchical structures within the built environment.
      - This order may roughly align with temporal, spatial and social structures of cycling behavior they affect. For example, specific cycling infrastructure is expected to affect route considerations more strongly, whereas land use or transport system presumably do not affect day-to-day activity considerations as much, but are more influential for location or ownership choices.
      - Network connectivity and quality are depicted as separate aspects of infrastructure, which in combination determine walkability and bikeability.
  - Social layers
    - MINDSPACE: for how policy influences individual behavior, see 1.3.4 (Dolan et al. 2010)
- Choices and outcomes
  - Active travel related discrete choices are depicted separately (boxes with arrows) from more aggregated active travel related outcomes to illustrate various key concepts of interest in active travel behavior. Walking related outcomes are in orange, cycling related outcomes in yellow, and physical activity related outcomes in green. For a systematic overview of choices and outcomes, see supplemental materials [here](https://www.researchgate.net/profile/Thomas_Goetschi/contributions).
